# A Biodegradable, Polymer-Supported Oxygen Atom Transfer Reagent

**DOI:** 10.3390/polym15092052

**Published:** 2023-04-26

**Authors:** Erin E. Ramey, Elizabeth L. Whitman, Cole E. Buller, James R. Tucker, Charles S. Jolly, Kjersti G. Oberle, Austin J. Becksvoort, Mark Turlington, Christopher R. Turlington

**Affiliations:** 1 Department of Chemistry and Biochemistry, Hope College, Holland, MI 49422, USA; 2 Department of Chemistry and Biochemistry, Berry College, Mount Berry, GA 30149, USA

**Keywords:** biodegradable polymers, oxygen atom transfer, organocatalytic ring-opening polymerization

## Abstract

Biodegradable polymers are desirable to mitigate the environmental impact of plastic waste in the environment. Over the past several decades, the development of organocatalytic ring-opening polymerization (OROP) has made the synthesis of many new types of biodegradable polymers possible. In this research article, the first example of an oxygen atom transfer reagent pendant on a biodegradable polymer backbone is reported. The monomers for the polycarbonate backbone are sourced from the biodegradable 2,2-bis(hydroxymethyl) propionic acid molecule, and an iodoaryl group is installed pendant to the cyclic monomer for post-polymerization modification into an iodosylaryl oxygen atom transfer reagent. The key I-O bond is characterized by XPS spectroscopy, and a test reaction to triphenylphosphine demonstrates the ability of the polymer to engage in an oxygen atom transfer reaction with a substrate.

## 1. Introduction

The development of biodegradable polymer backbones is of great interest due to the long lifetimes of many traditional polymer products and the alarming rate at which these non-biodegradable materials are being added to the environment. In 2010, for example, it was estimated that 4.8–12.7 million metric tons of plastic waste were added to the oceans, and the total amount of plastic waste in the oceans was predicted to increase by a factor of ten after an additional fifteen years [1]. To reduce their environmental impact, many researchers seek to develop environmentally friendly alternatives. Some promising strategies have been proposed, such as including cyclic ketene acetals as degradable additives in the radical polymerization of polyolefins [2,3], but matching the physical and material properties of the existing polymer products is challenging. Ideally, biodegradable polymer backbones could be developed as substitutes for the non-biodegradable products that dominate the packaging and consumer product industries.

The field of organocatalytic ring-opening polymerization (OROP) is an attractive strategy for the synthesis of biodegradable polymer backbones. In OROP, organic catalysts have been tailored for the rapid, controlled polymerization of many cyclic monomer classes, including but not limited to lactones (yielding polyesters), carbonates (polycarbonates), and phosphazenes (polyphosphazenes) [4]. Many degradation studies of such polymer backbones have been completed in acidic or basic media, and the lifetimes of the polymers vary greatly depending on the chemical structure of the polymers, ranging from minutes or hours to months and years [4]. These lifetimes are much more environmentally friendly than traditional plastics. Polymers that are synthesized from OROP have unique physical and chemical properties that promise applications in many fields, and such polymers have already been used in drug delivery, dissolving sutures, and manufacturing (polylactide cups and utensils) [5].

**Figure 1:**
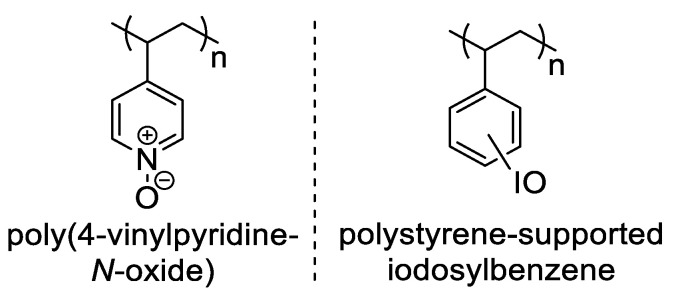
Two polymer-supported, oxygen atom transfer reagents that are not biodegradable.

The field of OROP is still relatively young, and many more applications are still waiting to be discovered. Our laboratory has had an interest in oxygen atom transfer (OAT) reagents for organic and organometallic transformations, and recently published a paper exploring the use of poly(4-vinylpyridine-*N*-oxide) as a polymer-supported OAT reagent (Figure 1) [6]. While the poly(4-vinylpyridine-*N*-oxide) was easily synthesized and could be recycled, it was not a strong oxidant, and the vinyl polymer backbone was non-biodegradable. This provided motivation to search for stronger oxidants and anchor them into a biodegradable polymer backbone. Iodosylarenes (ArIO) are strong OAT reagents, and their incorporation into non-biodegradable polystyrene supports is well known [7,8]. However, the inclusion of iodosylarene units into a biodegradable polymer backbone has not been reported.

**Figure 2:**
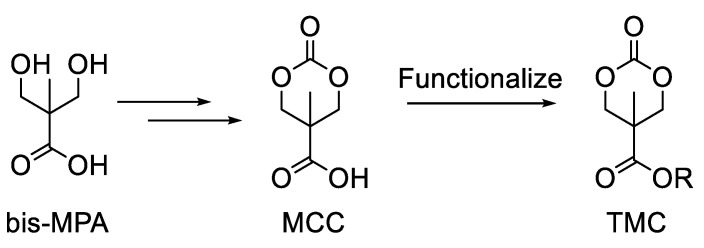
The biodegradable bis-MPA molecule for biodegradable monomer and polymer synthesis.

When considering options for a biodegradable polymer backbone, aliphatic polycarbonates are an attractive choice because of their biocompatibility and biodegradation properties [9,10]. In particular, polycarbonates derived from trimethylene carbonate (TMC) derivatives have been frequently employed for a wide variety of biomedical applications due to their low toxicity and tunable biodegradability [11]. Additionally, polycarbonates derived from TMC monomers can be readily constructed using OROP [12,13], and functionalized TMC monomers are efficiently accessed from the widely employed 5-methyl-5-carboxyl-1,3-dioxan-2-one (MCC) monomer (see Figure 2). MCC can be synthesized in large quantities, starting from the abundant and inexpensive 2,2-bis(hydroxymethyl)propionic acid (bis-MPA), which is also essentially non-toxic and biodegradable [14,15]. For these reasons, bis-MPA was chosen as a versatile and environmentally friendly precursor for the synthesis of a TMC monomer functionalized with an iodoaryl functional group. This article describes the synthesis, homopolymerization, and copolymerization of that monomer, along with the post-polymerization oxidation of the iodoaryl group to the reactive iodosylaryl group. Based on our knowledge, this represents the first example of a biodegradable, polymer-supported iodosylarene OAT reagent.

## 2. Materials and Methods

All reactions were performed under an atmosphere of dry nitrogen using standard drybox and Schlenk techniques, unless noted otherwise. Each solvent for polymerization reactions was stirred over calcium hydride for three days under nitrogen, and then transferred by vacuum distillation into a Chemglass air-free storage vessel (Vineland, NJ, USA) with activated 3 Å molecular sieves. Each solvent was degassed over three cycles of freeze–pump–thaw and taken into the glovebox. Benzyl alcohol was purchased from Acros Organics (Geel, Belgium) and purified by flash chromatography on silica gel using ethyl acetate as the eluent. The ethyl acetate was removed on a rotary evaporator, and the benzyl alcohol was transferred into a Chemglass air-free storage vessel with activated 3 Å molecular sieves. The benzyl alcohol was degassed over three cycles of freeze–pump–thaw and taken into the glovebox. 1,8-Diazabicyclo[5.4.0]undec-7-ene (DBU) was purchased from Millipore Sigma (Burlington, MA, USA) (puriss., ≥99.0%, catalog number 33482) and taken into the glovebox unopened and transferred under the nitrogen atmosphere to a Chemglass air-free storage vessel with activated 3 Å molecular sieves. N′-[3,5-Bis(trifluoromethyl)phenyl]-N-cyclohexylthiourea (TU) was synthesized according to the literature [16], and the thiourea catalyst was purified by flash chromatography on a silica gel column using 50/50 mixture of hexane and ethyl acetate as an eluent. The solvent was removed using rotary evaporator and the solid was dried under high vacuum at 40 °C overnight while stirring and then taken into the glovebox and stored under nitrogen. The cyclic carbonate monomer with a pendant benzyl group (Bn-TMC, benzyl 5-methyl-2-oxo-1,3-dioxane-5-carboxylate) was synthesized according to the literature [17]. The product was recrystallized from a concentrated solution of ethyl acetate by layering hexane on top. The crystalline material was isolated by filtration, washed with hexane, dried under vacuum, and then taken into the glovebox and stored under nitrogen. All other reagents were purchased and used as received. 

Analytical thin-layer chromatography (TLC) was performed on 200 μm silica gel plates from Millipore Sigma. Visualization was accomplished via UV light, and/or the use of potassium permanganate, followed by application of heat. Chromatography was performed using Silica Gel 60 (230–400 mesh) from Sorbent Technologies (Norcross, GA, USA). ^1^H, ^13^C, and ^31^P NMR spectra were recorded on either a JEOL 400 MHz spectrometer (Peabody, MA, USA) or a Bruker Avance III 400 MHz spectrometer (Billerica, MA, USA), and ^1^H NMR and ^13^C NMR chemical shifts were referenced to internal TMS standard set to 0 ppm or to residual ^1^H or the ^13^C of the deuterated solvents, respectively. NMR data are reported as follows: chemical shift, multiplicity (s = singlet, bs = broad singlet, d = doublet, t = triplet, q = quartet, quint = quintet, sext = sextet, m = multiplet, dd = doublet of doublets, dt = doublet of triplets, td = triplet of doublets, and qd = quartet of doublets), integration, and coupling constant (Hz). Elemental analyses were performed by Robertson Microlit Laboratories of Ledgewood, NJ. 

All GPC samples were prepared using ~3 mg of sample per 1 mL of THF and filtered through a 0.45 μm PTFE filter. THF was used as the mobile phase for all GPC instruments. The GPC-RI data were collected at Hope College on a Waters Alliance HPLC System (2695 Separation Module), equipped with Waters 5 µm Styragel columns (HR 4E, HR 3, and HR 4, MW linear range 200–400,000 g/mol, plus a guard column, and 0.300 mL/minute flow rate), and equipped with a Waters 2414 Refractive Index (RI) detector. The GPC-UC data with universal calibration were collected externally on a Waters APC separations module outfitted with three Waters Acquity columns (XT 45, XT 200, and XT 450a, MW linear range 200–400,000 g/mol, and 0.750 mL/minute flow rate) and a Malvern OMNISEC REVEAL detector (RI, Photodiode Array (PDA), and Viscometer). GPC-LS data were collected externally at UC Santa Barbra’s Materials Research Laboratory on a Waters Alliance HPLC System (2695 Separation Module) equipped with an Agilent PLgel 5 µm column (MiniMIX-D, 250 × 4.6 mm, MW linear range 200–400,000 g/mol, plus a guard column, and 0.375 mL/minute flow rate) equipped with a Wyatt DAWN HELEOS-II Multi-Angle Light Scattering detector (Goleta, CA, USA) (MALS, laser 663.1 nm), a Wyatt Optilab rEX RI detector, a Wyatt ViscoStar detector, and a Waters 2996 PDA detector (Milford, MA, USA). 

The iososylaryl samples were analyzed using the ThermoScientific Nexsa X-ray Photoelectron Spectrometer (Waltham, MA, USA) (XPS) with a hemispherical analyzer and monochromatic Al K*α* source (1486.7 eV). White gummy samples were received in sealed glass containers. A small amount (~1 mL) of chloroform (>99%) was added to each glass container to make the gummy samples “soft”. Then, the samples were taken out by means of a spatula and placed on conductive Cu tape. A thick layer of sample (>1 mm) was adhered to the Cu tape. Then, the sample holder was loaded into the entry-lock chamber and held under vacuum (<5 × 10^–6^ mbar) for ~45 min. Once the pressure in the entry-lock chamber reached ~3.5 × 10^–7^ mbar, the sample holder was transferred into the analysis chamber. The base pressure of the analysis chamber during the data acquisition was <2.4 × 10^–7^ mbar. The XPS survey spectra were collected using a pass energy of 150 eV, an energy step size of 1.0 eV, and a 20 ms/step dwell time. High-resolution spectra of C1s, O1s, and I3d core lines were collected using 50 eV pass-energy, 0.1 eV energy step size, and 150 ms/step dwell time. Recorded spectra were analyzed using ThermoAvantage^®^ v5.9922 software to extract qualitative information from the survey and high-resolution spectra.

**Synthesis of ArI-TMC (4-iodobenzyl 5-methyl-2-oxo-1,3-dioxane-5-carboxylate)**. 5-methyl-2-oxo-1,3-dioxane-5-carboxylic acid (MCC, 1.76 g, 10.94 mmol, 1 equiv) was dissolved in anhydrous THF (36.5 mL) in a 100 mL flame-dried round bottom flask under nitrogen atmosphere. Oxalyl chloride (0.926 mL, 10.94 mmol, 1 equiv) was added, followed by the addition of DMF (0.11 mL), which resulted in vigorous gas evolution. The reaction was stirred at room temperature for 1 h to generate the acid chloride intermediate. After 1 h, the solvent was removed by rotary evaporation, yielding the acid chloride as a pale-yellow oil. The acid chloride was redissolved in anhydrous THF (10 mL) and concentrated again by rotary evaporation. The acid chloride was then dissolved in anhydrous THF (27 mL) and placed under nitrogen atmosphere. In a flame-dried 50 mL pear flask, 4-iodobenzyl of alcohol (2.56 g, 10.94 mmol, 1 equiv) was dissolved in anhydrous THF (23 mL) and Et_3_N (1.68 mL, 12.03 mmol, 1.1 equiv) was added. This solution was then transferred to the reaction flask containing the acid chloride via cannula. The pear flask was rinsed with anhydrous THF (2 mL × 2) and each rinse was transferred to the reaction flask. The reaction was then stirred at room temperature for 20 h, after which it was filtered through Celite and rinsed with EtOAc. After concentration via rotary evaporation, the resulting crude reaction mixture was purified by flash silica gel column chromatography (100% hexanes to 50:50 hexanes/EtOAc) to yield the product as a white solid in 76% yield (3.13 g). R_f_ = 0.39 (2:1 hexanes/EtOAc). IR (neat, cm^−1^) 3049, 2928, 1724, 1457, 1238, 1171, and 1094. ^1^H NMR (400 MHz, CDCl_3_) δ 7.72 (d, 2H, J = 8.4 Hz), 7.09 (d, 2H, J = 8.4 Hz), 5.16 (s, 2H), 4.69 (d, 2H, J = 10.8 Hz), 4.21 (d, 2H, J = 11.2 Hz), and 1.32 (s, 3H). ^13^C NMR (100 MHz, CDCl_3_) δ 171.0, 147.5, 138.0, 134.5, 130.2, 94.7, 73.0, 67.3, 40.4, and 17.6. Anal. Calcd (found) for C_13_H_13_IO_5_: C, 41.51 (41.56); H, 3.48 (3.36); N, 0.00 (< 0.10). The compound was recrystallized before polymerization reactions by layering hexanes on top of a concentrated solution of the monomer in ethyl acetate.

**Homopolymerization of ArI-TMC**. A 3 mL vial with a screw cap and PTFE septum in the glovebox was charged with 1.6 mg (4.3 μmol, 8 mole %) of co-catalyst 1-(3,5-bis(trifluoromethyl)-phenyl)-3-cyclohexyl-2-thiourea (TU), 20.0 mg (0.053 mmol, 0.35 *M*) of monomer ArI-TMC, 0.150 mL toluene, and 6.6 μL of a benzyl alcohol initiator solution (0.080 *M* in toluene, 5.3 × 10^−4^ mmol), respectively. Then, 0.6 μL of a solution of co-catalyst 1,8-diazabicyclo[5.4.0]undec-7-ene (DBU, 1.3 *M* in toluene, 0.78 μmol, and 1.5 mole %) was added to begin the polymerization. The reaction mixture was stirred at room temperature for 2.5 h, and then the sample was taken out of the glovebox and quenched with a drop of acetic acid. The solvent was blown off, and the crude product was dried under high vacuum. The sample was then dissolved in *d*-chloroform and analyzed by ^1^H spectroscopy to determine the percent conversion (>95%). The NMR sample was dialyzed in 500 mL stirring THF for 2 days, at which point the sample was blown off, and then dried under high vacuum, yielding 10.0 mg (50%). ^1^H NMR (400 MHz, CDCl_3_) δ 7.65 (d, 2H, J = 8.0 Hz), 7.04 (d, 2H, J = 8.0 Hz), 5.07 (s, 2H), 4.26 (broad s, 4H), and 1.23 (s, 3H). GPC-RI (expected *M*_n_ = 37,600): *M*_n_ = 9900, *M*_w_ = 11,100, and *Đ* = 1.12. GPC-UC (expected *M*_n_ = 37,600): *M*_n_ = 40,300, *M*_w_ = 49,000, and *Đ* = 1.22.

**Copolymerization of 40% ArI-TMC and 60% Bn-TMC**. A 3 mL vial with a screw cap and PTFE septum in the glovebox was charged with 27.9 mg (0.075 mmol, 5 mole %) of co-catalyst TU, 226.3 mg (0.60 mmol, 0.40 *M*) of monomer ArI-TMC, 225.0 mg (0.90 mmol, 0.60 *M*) of monomer Bn-TMC, and 1.5 mL DCM, respectively. Once all reagents were dissolved, 1.6 μL of the initiator benzyl alcohol (0.015 mmol) and 11.2 μL of the co-catalyst DBU (0.075 mmol, 5 mole %) were added to begin the polymerization. The reaction mixture was stirred at room temperature for 4 h, and then the sample was taken out of the glovebox and quenched by adding a drop of acetic acid. The solvent was blown off, and the crude product was dried under high vacuum. The residue was dissolved in a minimal amount of chloroform, and the solution was added dropwise to 15 mL of vigorously stirring methanol in a 20 mL tared scintillation vial. The solvent was removed with a pipette, and the product was dried under high vacuum, yielding 433.9 mg (96%). ^1^H NMR (400 MHz, CDCl_3_) δ 7.66 (d, 2H, J = 7.4 Hz), 7.30 (s, 5H), 7.04 (d, 2H, J = 7.5 Hz), 5.14 (s, 2H), 5.06 (s, 2H), 4.28 (s, 8H), and 1.24 (s, 6H). ^13^C NMR (100 MHz, CDCl_3_) δ 171.9, 171.8, 154.3, 154.3, 137.7, 135.4, 135.1, 129.9, 128.6, 128.3, 128.0, 94.1, 77.4, 68.6, 67.1, 66.4, 46.6, and 17.4. GPC-LS (expected *M*_n_ = 30,200): *M*_n_ = 22,600, *M*_w_ = 26,900, and *Đ* = 1.19.

**Relative rates of polymerization (50 mole % ArI-TMC and 50 mole % Bn-TMC)**. A 3 mL vial with a screw cap and PTFE septum in the glovebox was charged with 3.3 mg (0.0088 mmol, 5 mole %) of co-catalyst TU, 66.7 mg (0.176 mmol) of monomer ArI-TMC, 44.0 mg (0.176 mmol) of monomer Bn-TMC, and 0.75 mL of deuterated-DCM, respectively. Once all reagents were dissolved, the solution was transferred to an NMR tube, 0.20 μL of the initiator benzyl alcohol (0.0018 mmol) and 1.3 μL of the co-catalyst DBU (0.0088 mmol, 5 mole %) were added to begin the polymerization. The reaction was monitored by ^1^H spectroscopy to determine the percent conversion of each monomer every 15 min. The percent conversion of Bn-TMC was monitored by tracking the disappearance of the 2-proton resonance at 5.121 ppm, using the aryl resonances of Bn-TMC monomer/polymer between 7.326 and 7.171 ppm as an internal standard calibrated to 5 protons (the monomer and polymer resonances for Bn-TMC overlap, but are distinct from other chemical species). The percent conversion of ArI-TMC was monitored by comparing the ratio of the 2-proton aryl resonance of the monomer (7.631 ppm) to the polymer (7.588 ppm).

**Synthesis of Iodosyl Copolymer by oxidation of poly(40%[ArI-TMC]-co-60%[Bn-TMC])**. A tared 20 mL scintillation vial that was open to the air was charged with 337.9 mg (0.45 mmol ArI) of poly(40%[ArI-TMC]-co-60%[Bn-TMC]). The copolymer was dissolved in 3.2 mL of carbon tetrachloride, and 3.3 mL of concentrated hydrochloric acid was added to the vial while stirring at 0 °C. An amount of 67.5 mg (0.55 mmol) of potassium chlorate was added at a pace of about 2 mg/min and the reaction mixture continued to be stirred at 0 °C for 3 h. The reaction was warmed to room temperature, and the liquid was removed with a pipette. The copolymer was washed with cold, diluted hydrochloric acid and cold water and dried under high vacuum for 30 min. An amount of 2.1 mL of 5 M NaOH was added dropwise to the 20 mL scintillation vial while stirring at 0 °C. After 30 min, 2.1 mL (0.12 mol) of water was added, and the reaction was stirred at room temperature overnight. The liquid was removed with a pipette, and the iodosyl copolymer was washed with water and ethyl ether, yielding 290.7 mg (85%). The sample was then dissolved in *d*-chloroform and analyzed by ^1^H NMR (400 MHz, CDCl_3_) δ 7.66 (broad d, 2H), 7.31 (m, 5H), 7.05 (broad d, 2H), 5.15 (s, 2H), 5.08 (s, 2H), 4.29 (s, 8H), and 1.25 (s, 6H). XPS Spectroscopy (eV): C(1*s*) core lines (284.8, 286.8, 289.2, and 290.7), O(1*s*) core lines (532.4, 533.9), and I(3*d*) core lines (620.9, 632.4).

**Triphenylphosphine substrate oxidation.** For triphenylphosphine oxidation, 20.0 mg of the iodosyl copolymer (0.026 mmol ArIO) was dissolved in *d*-chloroform and reacted with 50 mole % of triphenylphosphine based on the iodosylbenzene loading of the copolymer (3.2 mg triphenylphosphine, 0.013 mmol). The percent conversion was determined by ^31^P NMR spectroscopy, and the product identities were confirmed by authentic standards.

## 3. Results and Discussion

**Figure 3:**
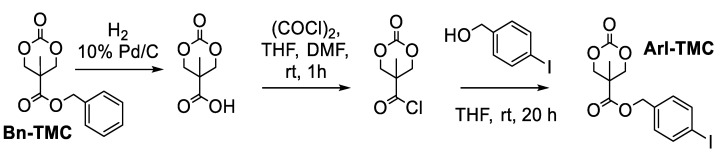
Synthesis of ArI-TMC, a cyclic trimethylene carbonate monomer with a pendant iodoaryl group.

The synthesis of the targeted iodoaryl-containing monomer first began using bis-MPA as a feedstock for the synthesis of the benzyl-containing cyclic trimethylene carbonate monomer (Bn-TMC, pictured in Figure 3), as previously reported [17]. With gram-scale quantities of Bn-TMC in hand, the benzyl group was readily removed by hydrogenation [17], and the resulting carboxylic acid was transformed into the corresponding acid chloride via treatment with oxalyl chloride and DMF. This intermediate was then immediately treated with 4-iodobenzyl alcohol to produce the desired cyclic trimethylene carbonate with a pendant iodoaryl functional group (ArI-TMC, Figure 3). The synthesis of ArI-TMC was completed on a multigram scale with good yield when using flame-dried glassware, anhydrous solvents, and cannula transfer techniques. The product was characterized by ^1^H and ^13^C NMR spectroscopy and was found to be analytically pure by elemental analysis. Recrystallization of the ArI-TMC monomer by layering hexanes on top of a concentrated solution in ethyl acetate yielded material that was suitable for polymerization reactions.

**Figure 4:**
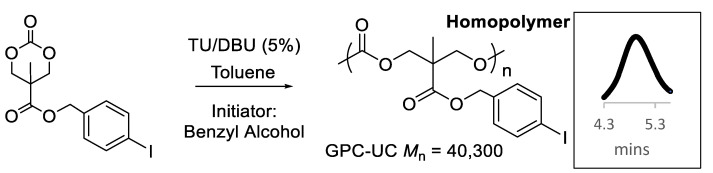
Homopolymerization of the ArI-TMC monomer and the polymer peak in the GPC-UC spectrum (inset).

Following the synthesis of the ArI-TMC monomer, homopolymerization of the monomer was attempted in toluene. A classic organic co-catalyst combination, the H-bonding N′-[3,5-Bis(trifluoromethyl)phenyl]-N-cyclohexylthiourea (TU), and the strong base 1,8-Diazabicyclo[5.4.0]undec-7-ene (DBU) were selected because of their good speed and control of the polymerization reaction [12]. When using benzyl alcohol as an initiator, the homopolymerization of ArI-TMC in toluene with the organic co-catalysts DBU and TU was successful, with high conversion to the polymer within a couple of hours (Figure 4). The percent conversion was determined to be greater than 95% using ^1^H NMR spectroscopy. The polymer was purified by dialysis, yielding a clean product.

Gel permeation chromatography with a refractive index detector (GPC-RI) revealed a single peak with a low dispersity, *Đ* = 1.12, which suggested that the homopolymerization had excellent control. However, the average molecular weight (*M*_n_) of the polymer was 9900 Daltons, which was much lower than expected for the targeted degree of polymerization and the percent conversion (37,600 Daltons). The calibration curve for the GPC-RI measurements was constructed from polystyrene standards. It has been shown for other polymers with heavy pendant groups, such as metal complexes, that GPC-RI underestimates the molecular weight when using common polymer standards such as polystyrene or poly(methyl methacrylate) [18,19,20]. Because of the heavy mass of the iodine atom, universal calibration (GPC-UC) was used, which couples measurements from a viscometer to the calibration curve to obtain an absolute determination of molecular weights [21]. In this case, a UV–visible detector, viscometer, and polystyrene standards were used to complete the GPC-UC experiment. Gratifyingly, the GPC-UC revealed an *M*_n_ of 40,300 Daltons, which was very close to the expected *M*_n_ (37,600 Daltons). The heavy iodine atom that was present in the pendant aryl group was likely the cause of the low mass reading by GPC-RI.

**Figure 5:**
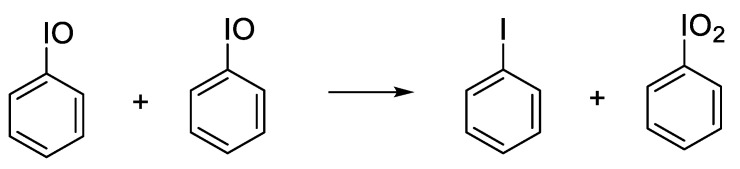
Disproportionation reaction of iodosylarenes. The ArIO_2_ product is not a strong oxygen atom transfer reagent.

**Figure 6:**
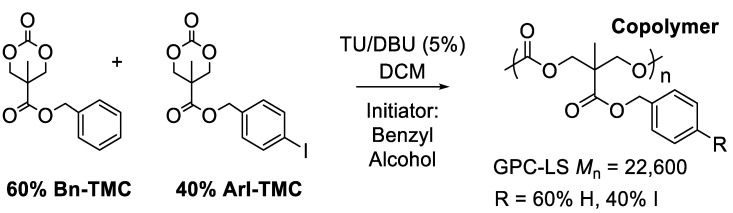
Copolymerization of Bn-TMC monomer and ArI-TMC monomer.

The successful homopolymerization demonstrated that the ArI-TMC monomer could be efficiently polymerized with the TU/DBU catalyst system. With this knowledge, we turned our attention to preparing a copolymer composed of ArI-TMC and Bn-TMC, as we deemed it would be prudent to space out the iodoaryl groups due to the disproportionation reaction of iodosylarene units becoming kinetically significant at higher concentrations (Figure 5) [22]. We reasoned that spacing out the iodoaryl groups in the copolymer could slow down disproportionation once the iodoaryl groups were oxidized to iodosylaryl groups, the targeted oxygen atom transfer (OAT) reagent. Accordingly, a random copolymer was synthesized with 60 mole percent Bn-TMC and 40 mole percent ArI-TMC, using the TU/DBU cocatalyst combination with benzyl alcohol initiator and dichloromethane (DCM) as the reaction solvent (Figure 6). DCM dissolved the two different monomers more efficiently than toluene, so that both monomers fully dissolved after several minutes of stirring. The initiator was then added to the homogeneous solution to start the polymerization reaction, and the reaction was run for four hours. The product was purified by dialysis. For molecular weight determination of the homopolymer, we opted to employ gel permeation chromatography with a multi-angle light scattering detector (GPC-LS), as GPC-LS is considered the most accurate method for the absolute determination of the molecular weight [23]. The GPC-LS spectrum showed a single polymer peak near the expected molecular weight (expected *M*_n_ = 30,200, actual *M*_n_ = 22,600). The copolymer also had a low dispersity, *Đ* = 1.19. Additionally, the product was characterized by ^1^H and ^13^C NMR spectroscopy.

**Figure 7:**
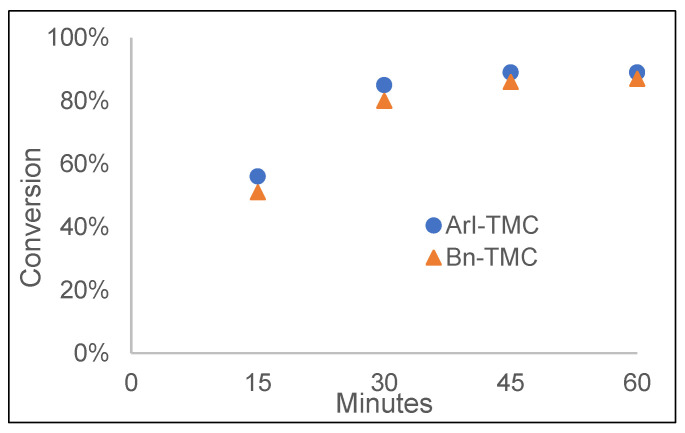
Reaction rates for the copolymerization of 50% Bn-TMC monomer and 50% ArI-TMC monomer.

To determine if the copolymerization was more random in nature or more gradient, a second copolymerization was attempted in deuterated-DCM, and the percent conversion of each monomer was traced over the course of the reaction by ^1^H NMR spectroscopy. A 50/50 mole ratio of ArI-TMC and Bn-TMC was used to simplify the analysis. It was found that the two monomers polymerized at very similar rates (Figure 7). For example, at the first data point in the reaction (15 min), the percent conversion of Bn-TMC was 51%, while the percent conversion of ArI-TMC was 56%. The two percent conversions for the two monomers were also similar after 30 min, when Bn-TMC was 80% and ArI-TMC was 85%. The two monomers also finished with similar percentage conversions after 60 min (87% for Bn-TMC and 89% for ArI-TMC). This led to the conclusion that the two monomers polymerized at very similar rates, which would suggest a random copolymer product as opposed to a gradient copolymer product. A random copolymer was desirable to better space out the iodoaryl groups and prevent disproportionation when the copolymer was oxidized to iodosylaryl groups. It was not surprising that the rates for polymerization of the two monomers were similar, as the only difference between ArI-TMC and Bn-TMC was the iodine atom in place of a hydrogen atom on the pendant aryl group.

**Figure 8:**
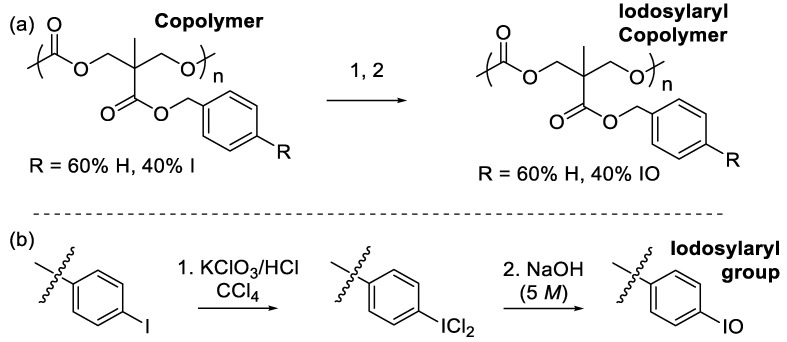
Oxidation of the iodoaryl groups to iodosylaryl groups in the copolymer. (**a**) Overall oxidation of the copolymer. (**b**) Reaction sequence occurring at the iodoaryl groups.

After isolating a purified sample of the random copolymer (40% ArI-TMC and 60% Bn-TMC), we turned our attention to the oxidation of the iodoaryl groups to the active iodosylaryl OAT reagents (Figure 8a). To do this, a two-step reaction was employed (Figure 8b) [24]. First, the iodo groups were oxidized to iodo(dichloride) groups using potassium chlorate and hydrochloric acid in carbon tetrachloride. After decanting the solvent and washing the iodo(dichloride) intermediate, the intermediate was immediately treated with 5 *M* sodium hydroxide to convert it to the iodosylaryl product. The product copolymer had the characteristic yellow tint of an iodosyl group, which was promising. However, the ^1^H NMR spectrum of the copolymer was essentially unchanged after the oxidation reaction, even though the peaks in the spectrum had broadened. The product of the oxidation reaction, therefore, required further characterization to elucidate its identity.

To elucidate the identity of the product, XPS spectroscopy was used to analyze the percentage composition of the product and also the oxidation state of iodine. For XPS spectroscopy, the percentage composition is based on the atomic percentage and is calculated from non-hydrogen atoms. To calculate the expected atomic percentages of the iodosylaryl copolymer, the molecular formulas of each unit were calculated without hydrogen and weighted for the appropriate percentage to gain a representative formula (0.60 × C_13_O_5_ + 0.40 × C_13_O_5_IO = C_13_O_5_I_0.4_O_0.4_). For this formula, the expected atomic percentages were calculated for C, O, and I (Table 1). The actual percentages found by XPS spectroscopy were in very close agreement for all three atoms. Carbon and oxygen only differed by 0.8%, and iodine was found to be 2.2%, which was very close to the calculated percentage of 2.1%. The atomic percentages provided good evidence that the oxidation of iodo groups to iodosyl units was successful.

**Figure 9:**
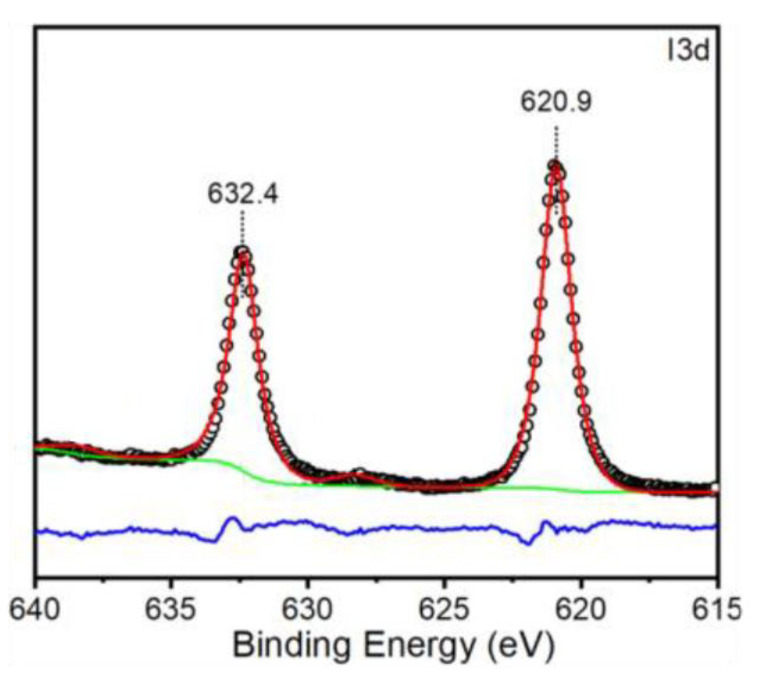
Expansion of the XPS region showing the resonance for iodine in the iodosylaryl copolymer.

Even better evidence was obtained by analyzing the peaks for iodine in the XPS spectrum. Because of the spin orbit coupling, each iodine species will appear as a 2:3 doublet represented by the I3d_3/2_ spin state and the I3d_5/2_ spin state, respectively. Only a single doublet was observed, which means that only one iodine species was present in the copolymer (Figure 9). Furthermore, the binding energy of the I3d_5/2_ band (620.9 eV) was closer to the +3 oxidation state reported for ICl_3_ (621.5 eV) than the 0 oxidation state reported for I_2_ (619.9 eV) [25,26], suggesting that iodine was closest to the +3 oxidation state and was coordinated to a highly electronegative atom, such as oxygen (see Table 2 for the literature that reported binding values for iodine). The percent composition, along with the high binding energy of iodine, all pointed to a successful synthesis of the biodegradable, polymer-supported oxygen atom transfer agent.

**Figure 10:**
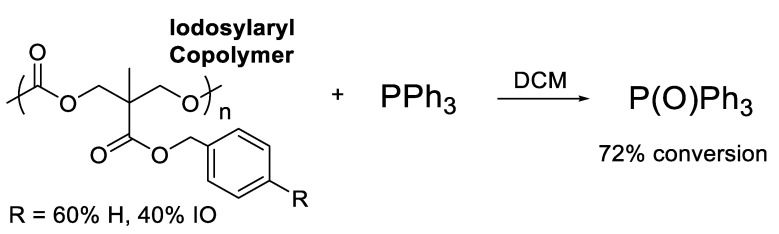
Reaction testing the oxidizing capability of the iodosylaryl copolymer.

The iodosyl copolymer was tested in an OAT reaction to give a preliminary judgment of its efficacy. The iodosyl copolymer was dissolved in deuterated chloroform and reacted at room temperature with 0.5 equivalents of triphenylphosphine relative to the iodosyl loading of the copolymer (Figure 10). The percent conversion to triphenylphosphine oxide was determined to be 72% by ^31^P NMR spectroscopy. The product was identified by comparison with the ^31^P NMR spectrum of a known sample of triphenylphosphine oxide. The oxidizing ability of the iodosyl copolymer was found to be much better than poly(4-vinylpyridine-*N*-oxide), which typically oxidized phosphines and phosphates to their respective oxides with less than 20% conversion, even with prolonged heating at 110 °C [6]. The result with the iodosyl copolymer demonstrated a clear improvement over previous work in our group and showed promise that the iodosyl copolymer could be useful in oxygen atom transfer reactions.

## 4. Conclusions

A new cyclic trimethylene carbonate monomer with a pendant iodoaryl group was synthesized. The monomer was suitable for homopolymerization reactions and also for a copolymerization reaction with a cyclic trimethylene carbonate monomer with a pendant benzyl group. The copolymer that was synthesized from the two monomers was functionalized post-polymerization to convert the iodoaryl groups to iodosylaryl groups, the active oxygen atom transfer reagent. The presence of the iodosylaryl groups was confirmed by XPS spectroscopy. Preliminary tests with triphenylphosphine show that the material can be used as a polymer-supported, oxygen atom transfer reagent. The polycarbonate backbone is biodegradable, making this the first example of a biodegradable, polymer-supported oxygen atom transfer reagent. Future directions include testing the reagent in the oxidation of organic and inorganic substrates and determining if the copolymer can be recycled.

## Figures and Tables

**Table 1:** Atomic percentages of the iodosylaryl copolymer found by XPS spectroscopy.

Element	Calcd. Atomic %	Actual Atomic %
Carbon	69.1	68.3
Oxygen	28.7	29.5
Iodine	2.1	2.2

**Table 2:** Literature-reported binding energy values for iodine.

Oxidation State	Compound	Binding Energy (eV)	Reference
−1	KI	619.3	[25]
0	I_2_	619.9	[26]
+3	ICl_3_	621.5	[26]
+5	KIO_3_	626.8	[25]
+7	KIO_4_	623.9	[25]

## Data Availability

The data presented in this study are available in the supplementary materials.

## Supplementary Materials

All NMR spectra of monomers and polymers (^1^H, ^13^C, and ^31^P), GPC traces of polymers, and XPS spectra are available and can be downloaded at the following: https://www.mdpi.com/article/10.3390/polym15092052/s1.
