# Supplementary material for: A Biodegradable, Polymer-Supported Oxygen Atom Transfer Reagent
Source: Polymers (Basel). 2023 Apr 26;15(9):2052. doi: 10.3390/polym15092052 (PMC10181130; doi:10.3390/polym15092052)

Supporting Information

A Biodegradable, Polymer-Supported Oxygen Atom Transfer Reagent

Erin E. Ramey [1], Elizabeth L. Whitman [2], Cole E. Buller [1], James R. Tucker [2], Charles S. Jolly [2], Kjersti G. Oberle [1], Austin J. Becksvoort [1], Mark Turlington [2] and Christopher R. Turlington [1,]*

1 Department of Chemistry and Biochemistry, Hope College, Holland, MI 49422, USA

2 Department of Chemistry and Biochemistry, Berry College, Mount Berry, GA 30149, USA

* Correspondence: turlington@hope.edu

Contents

I. NMR, GPC, and XPS Spectra 2
  A. ArI-TMC monomer 2
  B. ArI-TMC homopolymer 3
  C. Copolymer of 40% ArI-TMC and 60% Bn-TMC 5
  D. Iodosyl copolymer 7
II. Relative Polymerization Rates (50% ArI-TMC and 50% Bn-TMC) 9
III. Triphenylphosphine Oxidation 13

I. NMR, GPC, and XPS Spectra

A. ArI-TMC monomer

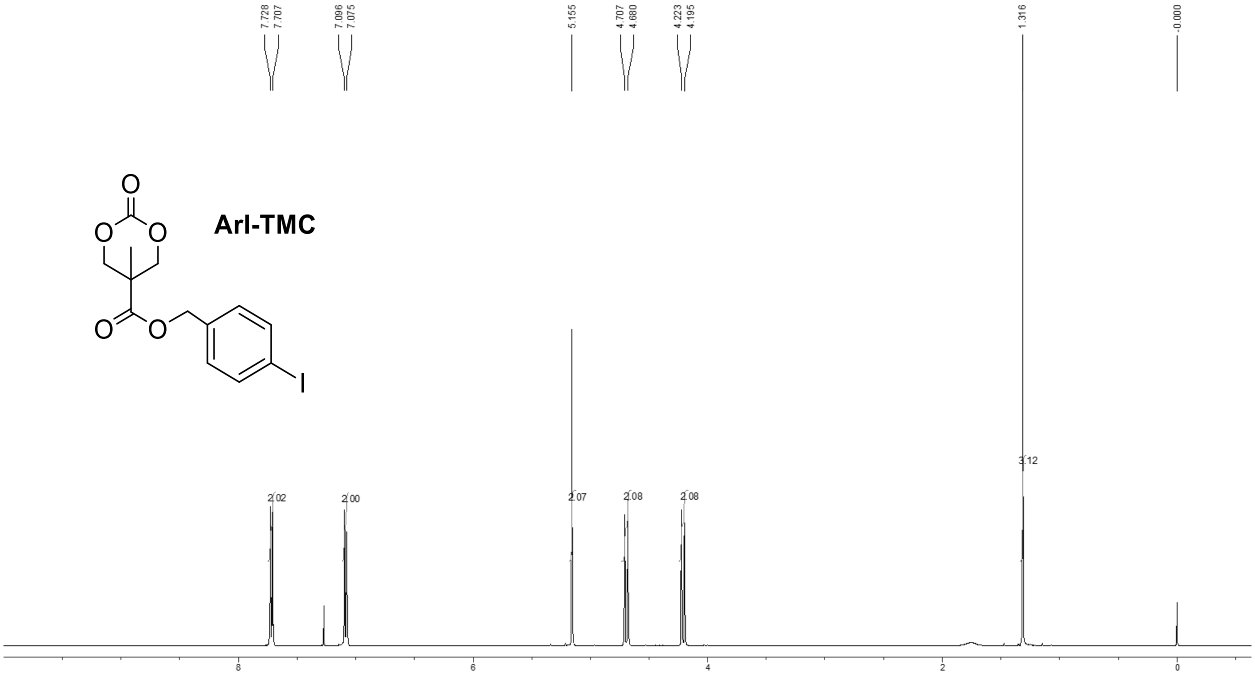

Figure S1. The ^{1}H NMR spectrum ($CDCl_3$) of ArI-TMC (4-iodobenzyl 5-methyl-2-oxo-1,3-dioxane-5-carboxylate).

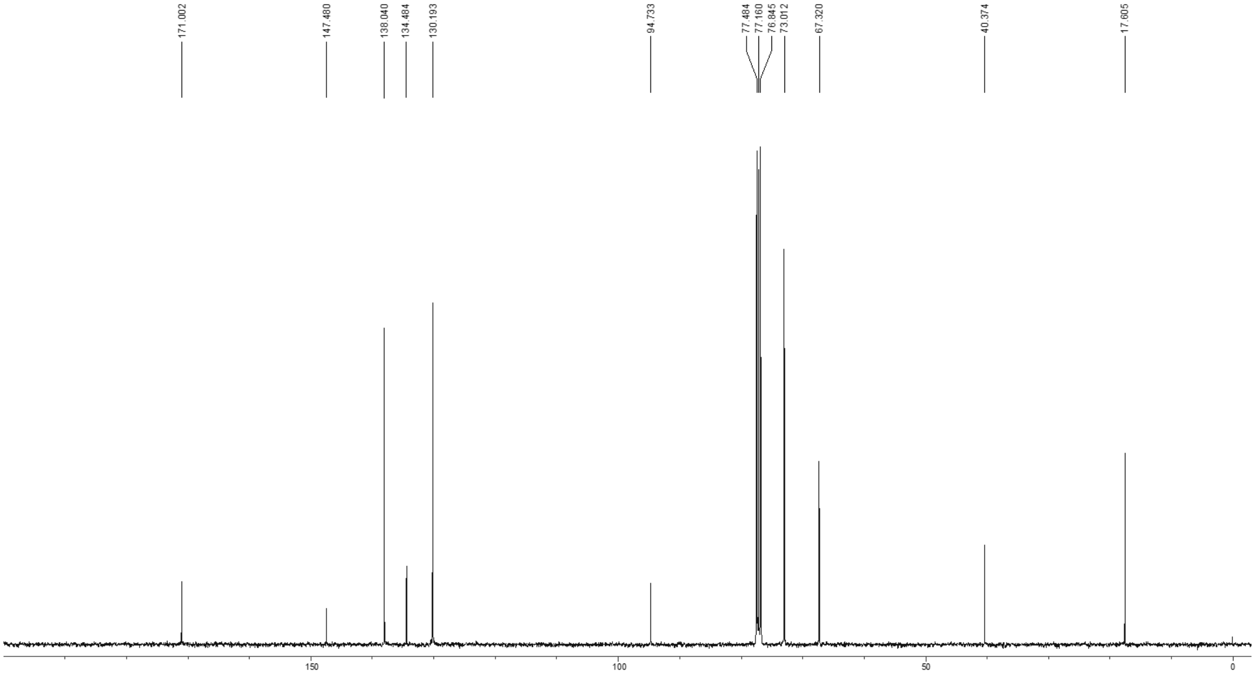

Figure S2. The ^{13}C NMR spectrum ($CDCl_3$) of ArI-TMC.

B. ArI-TMC homopolymer

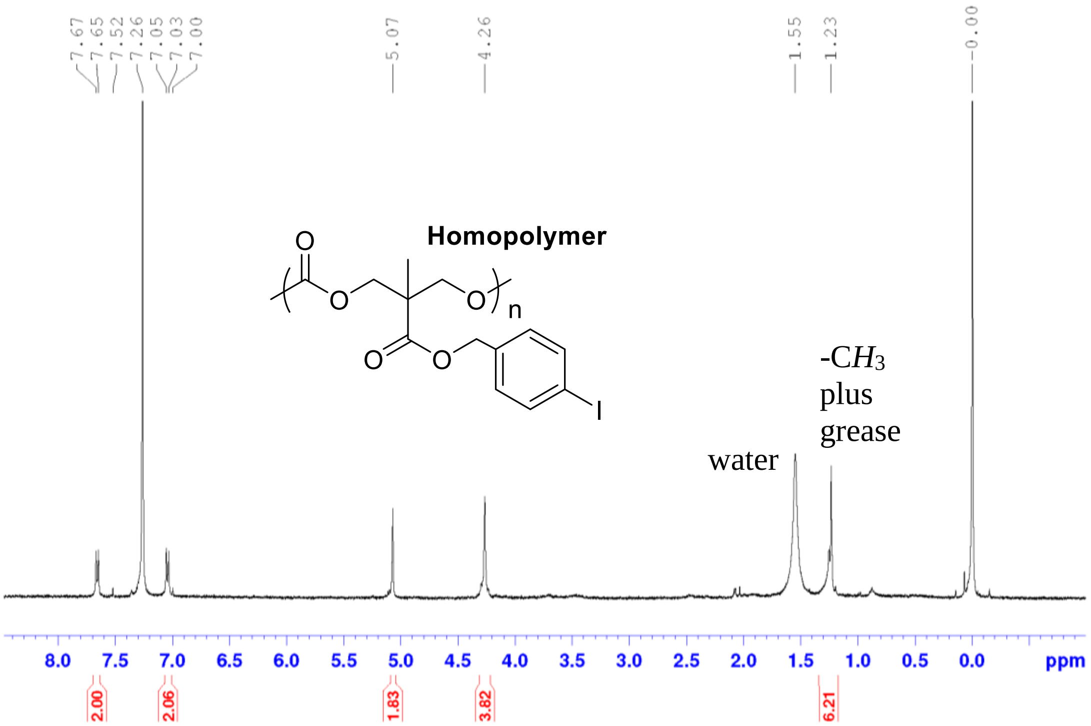

Figure S3. The ^{1}H NMR spectrum ($CDCl_3$) of the ArI-TMC homopolymer.

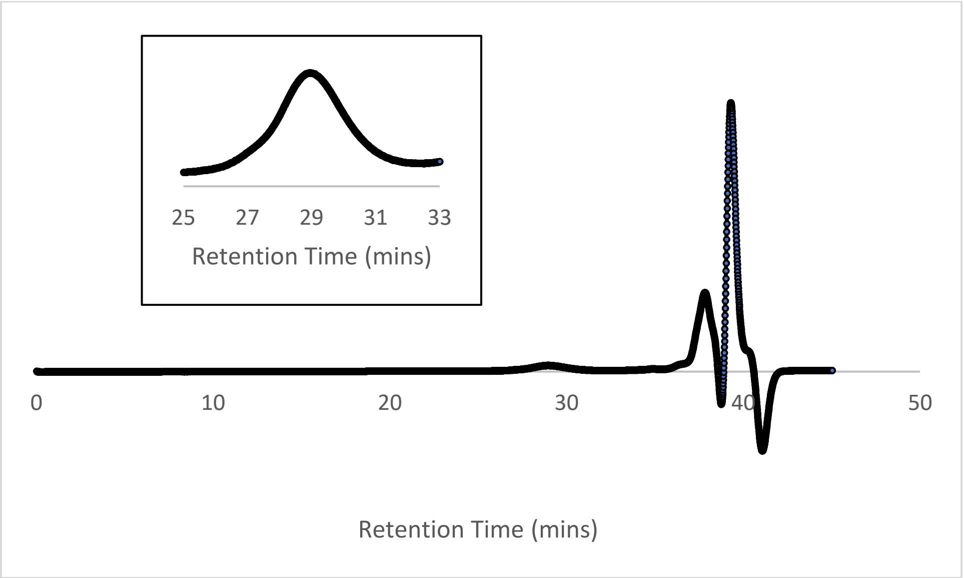

Figure S4. The GPC-RI spectrum (THF) of the ArI-TMC homopolymer. The inset shows an expanded view of the polymer peak. A refractive index detector (RI) was used, and the molecular weight was calculated by comparison to known polystyrene standards. GPC-RI (expected M_n=37,600): M_n=9,900, M_w=11,074, $Đ$= 1.12.

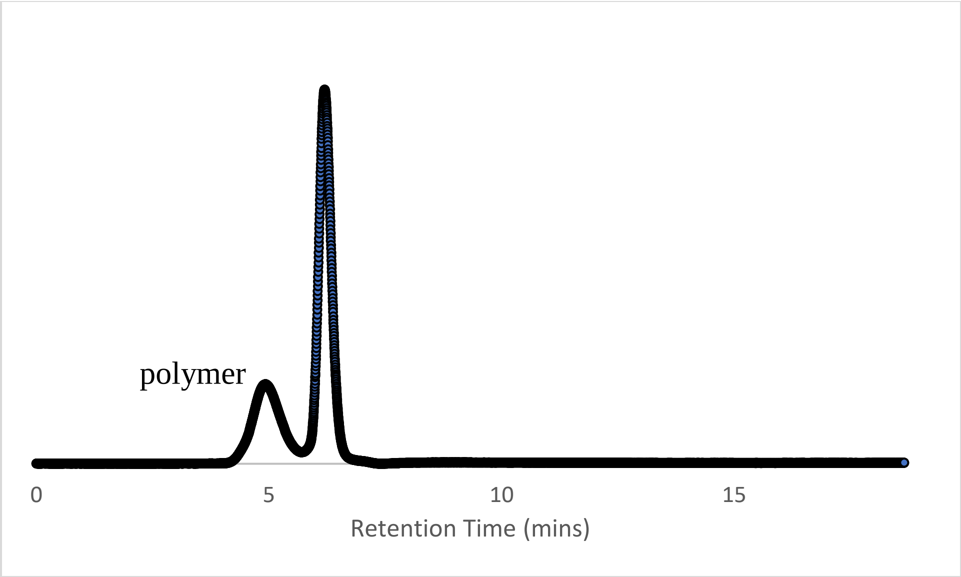

Figure S5. The GPC-UC (UC = Universal Calibration) spectrum in THF of the ArI-TMC homopolymer. A UV-visible detector (UV) was used, and the molecular weight was calculated by a Universal Calibration method using UV and Viscometer detectors with known polystyrene standards. GPC-UC (expected M_n=37,600): M_n=40,300, M_w=49,000, $Đ$= 1.22.

C. Copolymer of 40% ArI-TMC and 60% Bn-TMC

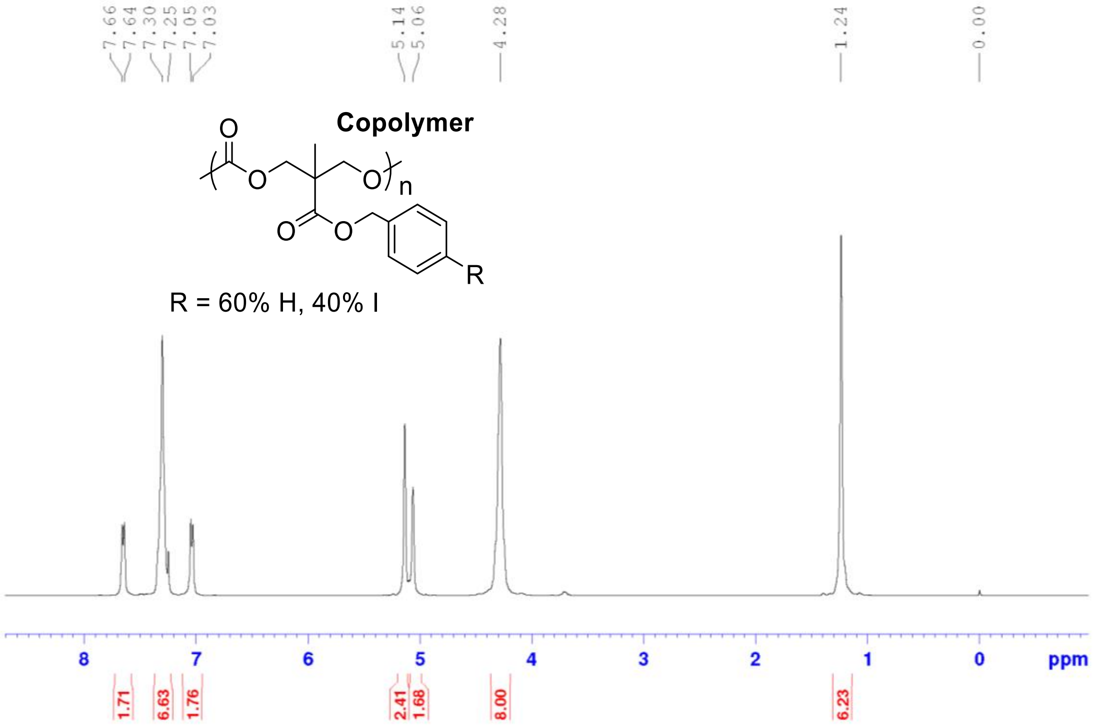

Figure S6. The ^{1}H NMR spectrum ($CDCl_3$) of the copolymer from polymerization of 40% ArI-TMC and 60% Bn-TMC.

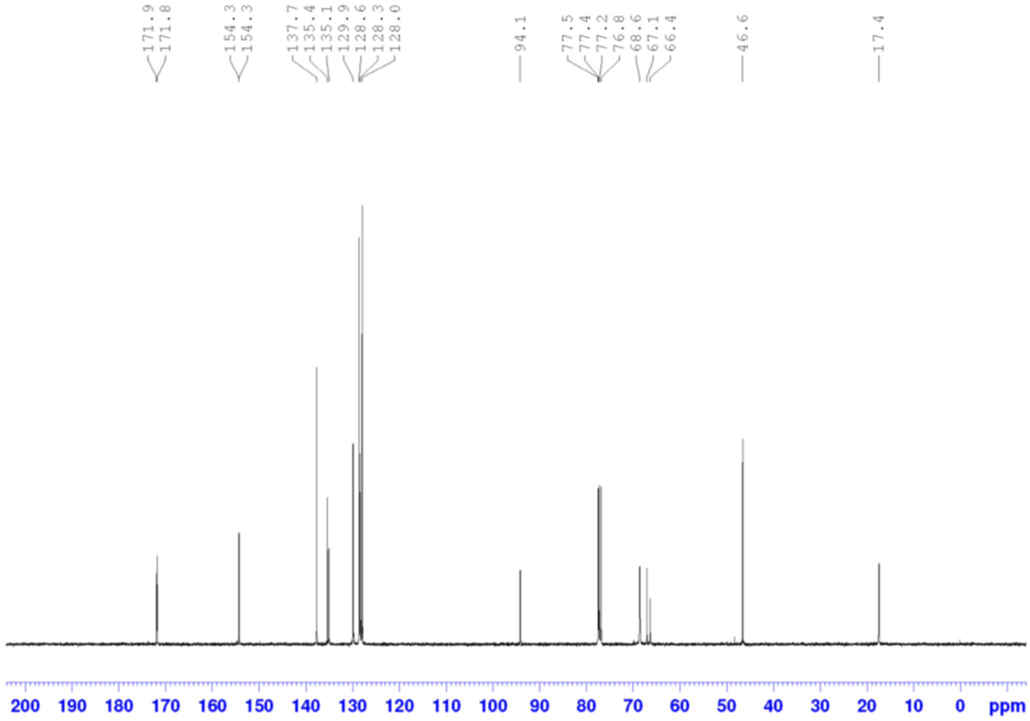

Figure S7. The ^{13}C NMR spectrum ($CDCl_3$) of the copolymer from polymerization of 40% ArI-TMC and 60% Bn-TMC.

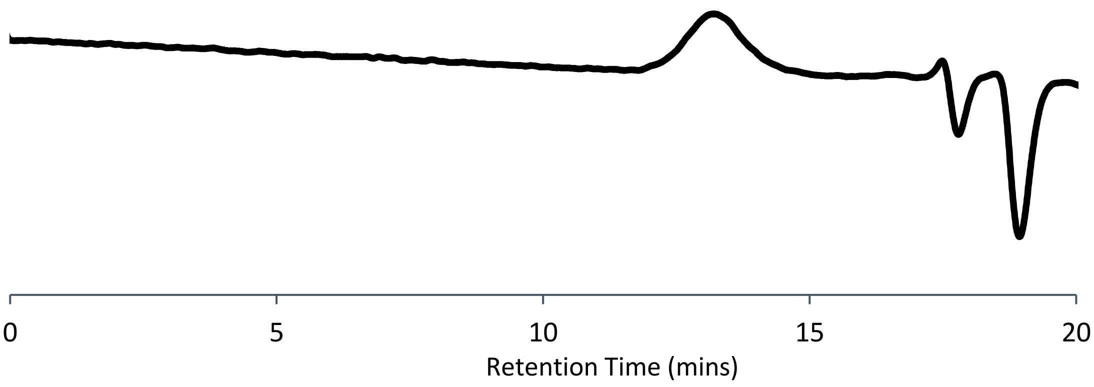

Figure S8. The GPC-LS spectrum (THF) of the copolymerization of 40% ArI-TMC and 60% Bn-TMC. GPC-LS (expected M_n=30,200): M_n= 22,600, M_w= 26,900, $Đ$= 1.19.

D. Iodosyl Copolymer

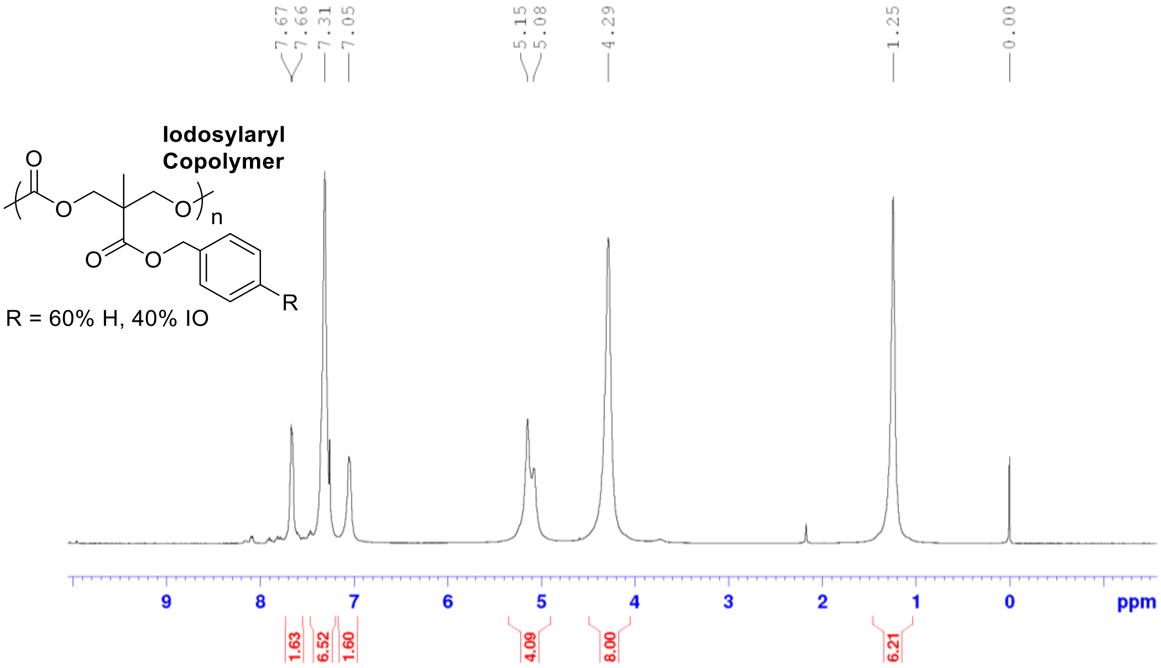

Figure S9. The ^{1}H NMR spectrum ($CDCl_3$) of the oxidation of poly(40%[ArI-TMC]-co-60%[Bn-TMC]) to the iodosyl copolymer.

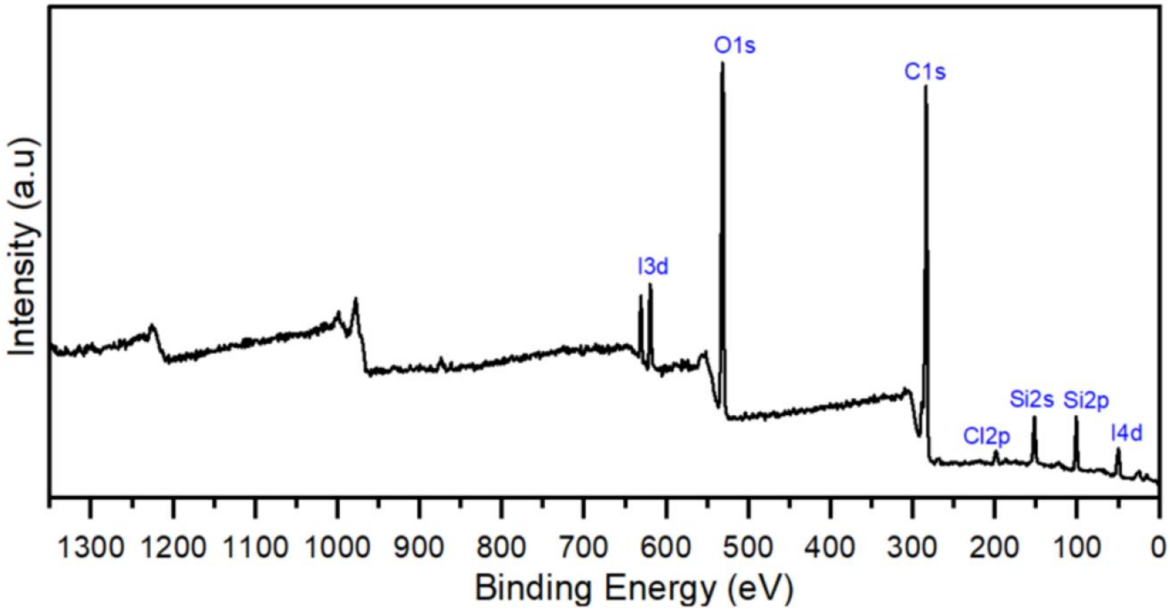

Figure S10. A survey XPS scan of the iodosyl copolymer. Iodine, oxygen, and carbon are detected. Chlorine is detected, but it is residual from the chloroform solvent used to prep the sample. Silicon is detected, but silicon is a common contaminant when storing samples in glass vials.

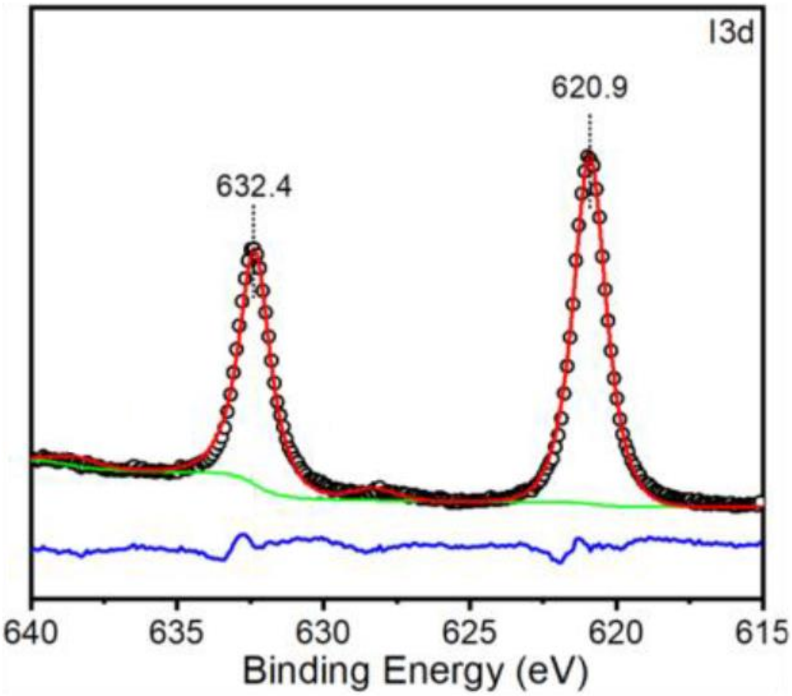

Figure S11. A high resolution XPS scan of the $I3d_{5/2}$ band (620.9 eV) and the $I3d_{3/2}$ band (632.4 eV), showing only a single iodine species was present. The $I3d_{5/2}$ band was closer to the +3 oxidation state reported for ICl_3 (621.5 eV) than the 0 oxidation state reported for I_2 (619.9 eV), suggesting that iodine was closest to the +3 oxidation state and was coordinated to a highly electronegative atom, such as oxygen.

II. Relative Polymerization Rates (50% ArI-TMC and 50% Bn-TMC)

15 minutes

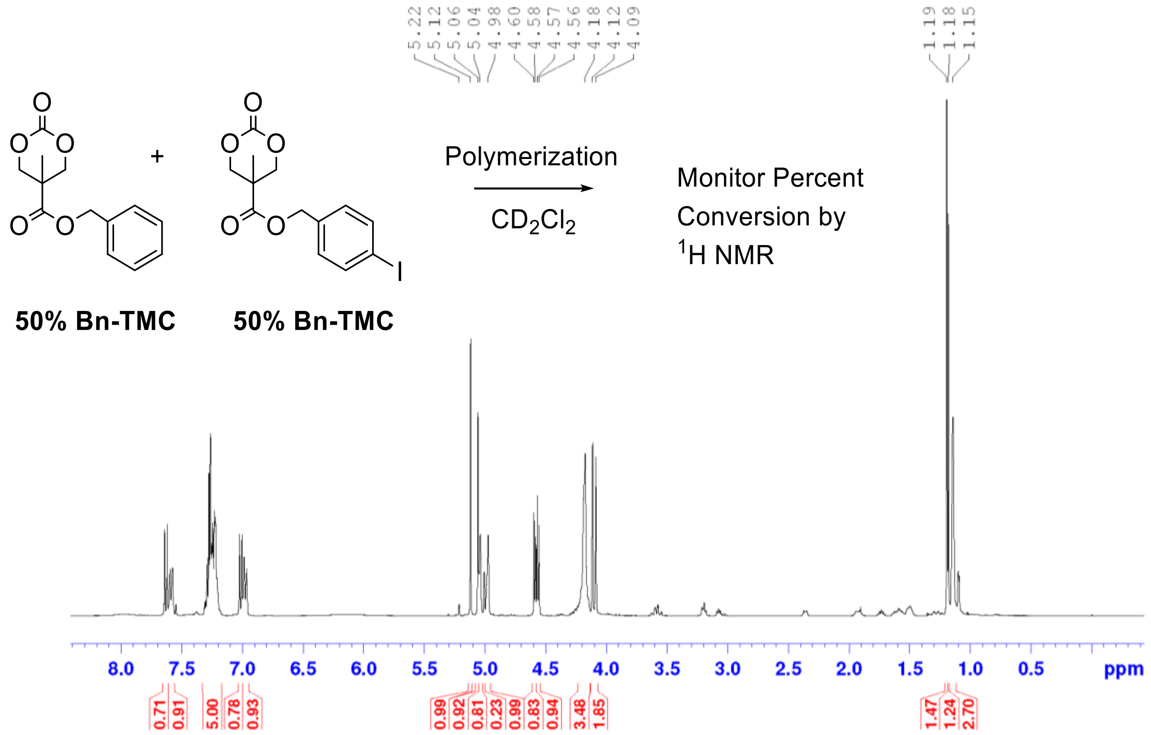

Figure S12a. The ^{1}H NMR spectrum (CD_2Cl_2) of the polymerization of 50% ArI-TMC and 50% Bn-TMC at 15 minutes of reaction time.

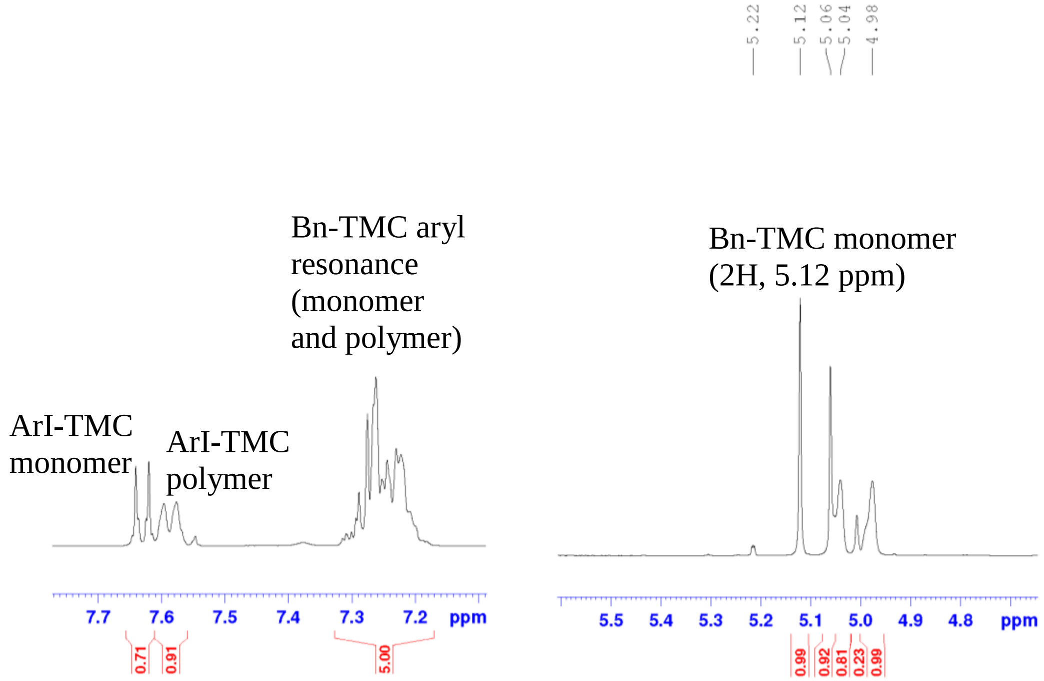

Figure S12b. Expansions of the ^{1}H NMR spectrum (CD_2Cl_2) at 15 minutes of reaction time. The percent conversion of ArI-TMC was 56% and the percent conversion of Bn-TMC was 51%.

30 minutes

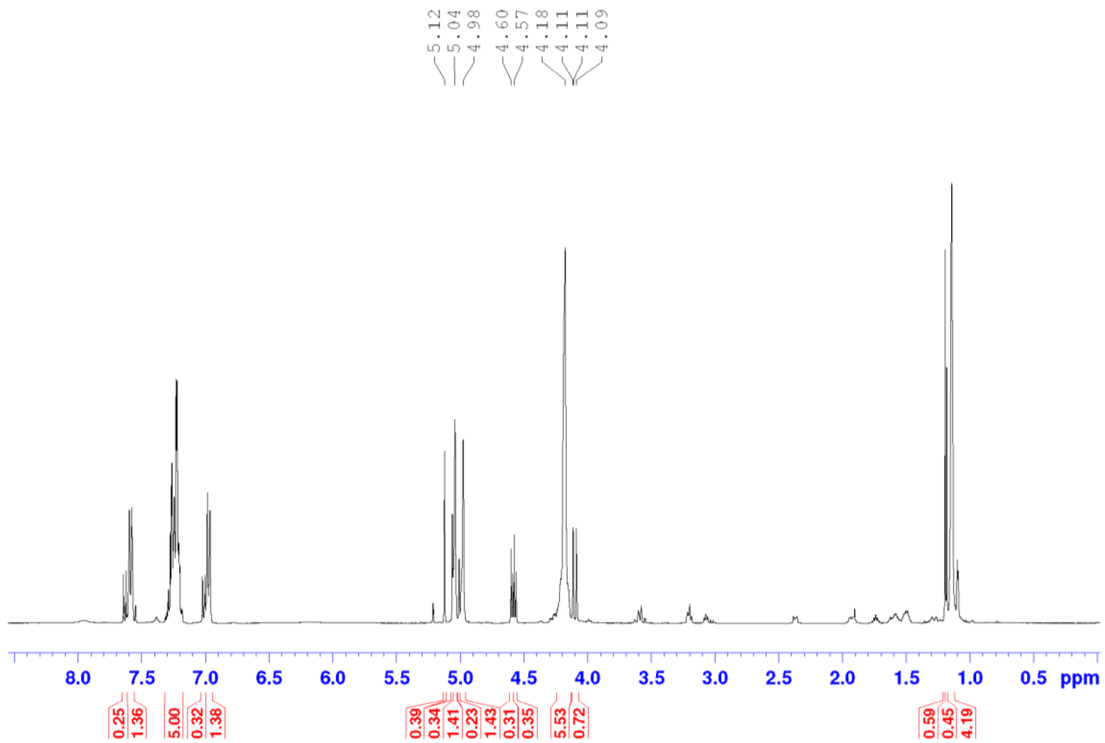

Figure S13a. The ^{1}H NMR spectrum (CD_2Cl_2) of the polymerization of 50% ArI-TMC and 50% Bn-TMC at 30 minutes of reaction time.

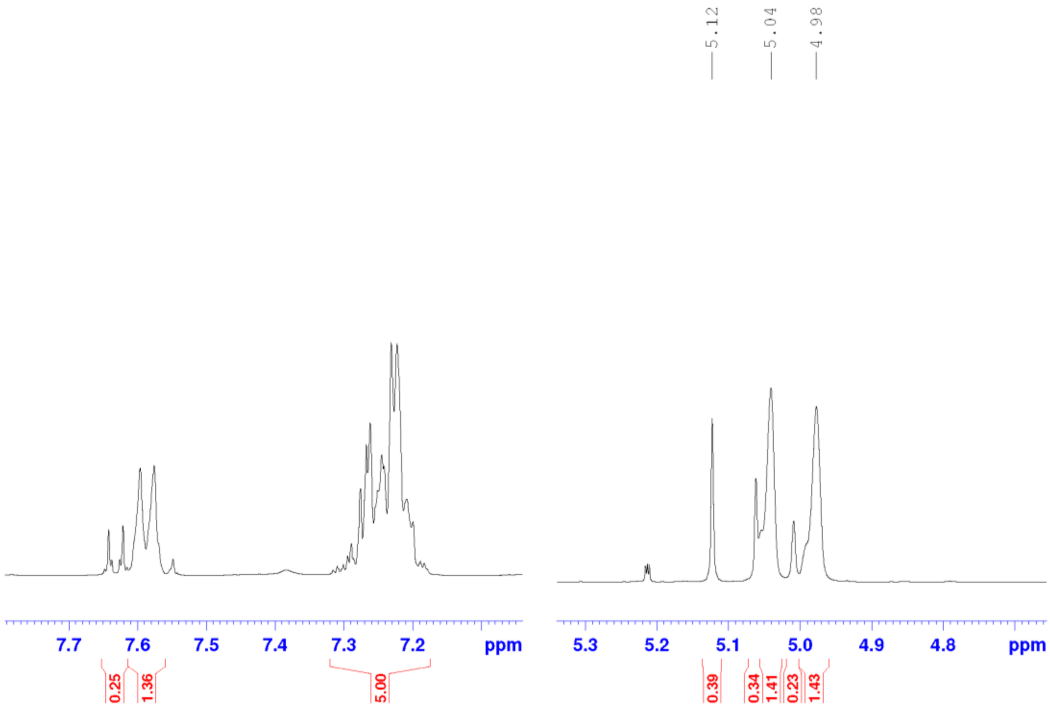

Figure S13b. Expansions of the ^{1}H NMR spectrum (CD_2Cl_2) at 30 minutes of reaction time. The percent conversion of ArI-TMC was 85% and the percent conversion of Bn-TMC was 80%.

45 minutes

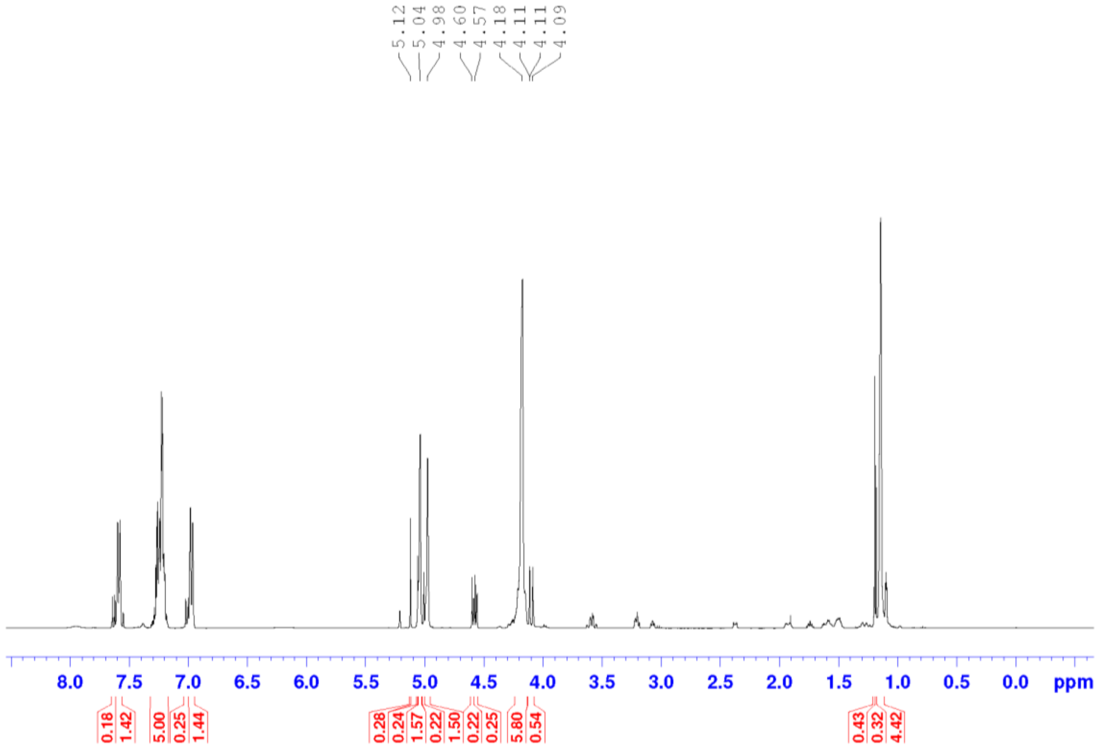

Figure S14a. The ^{1}H NMR spectrum (CD_2Cl_2) of the polymerization of 50% ArI-TMC and 50% Bn-TMC at 45 minutes of reaction time.

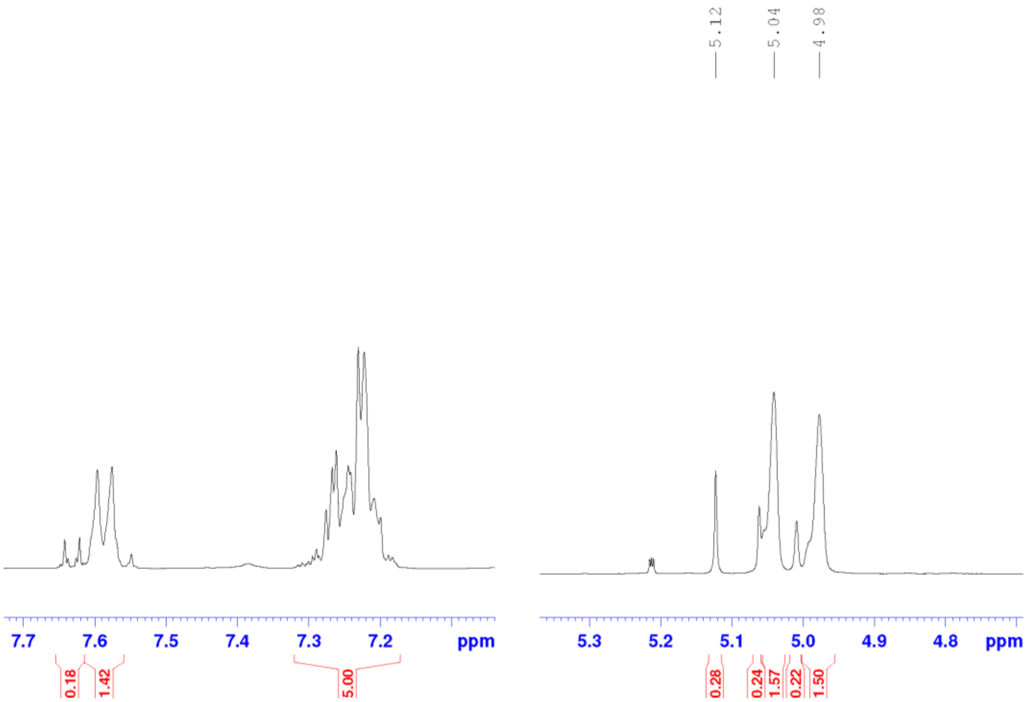

Figure S14b. Expansions of the ^{1}H NMR spectrum (CD_2Cl_2) at 45 minutes of reaction time. The percent conversion of ArI-TMC was 89% and the percent conversion of Bn-TMC was 86%.

60 minutes

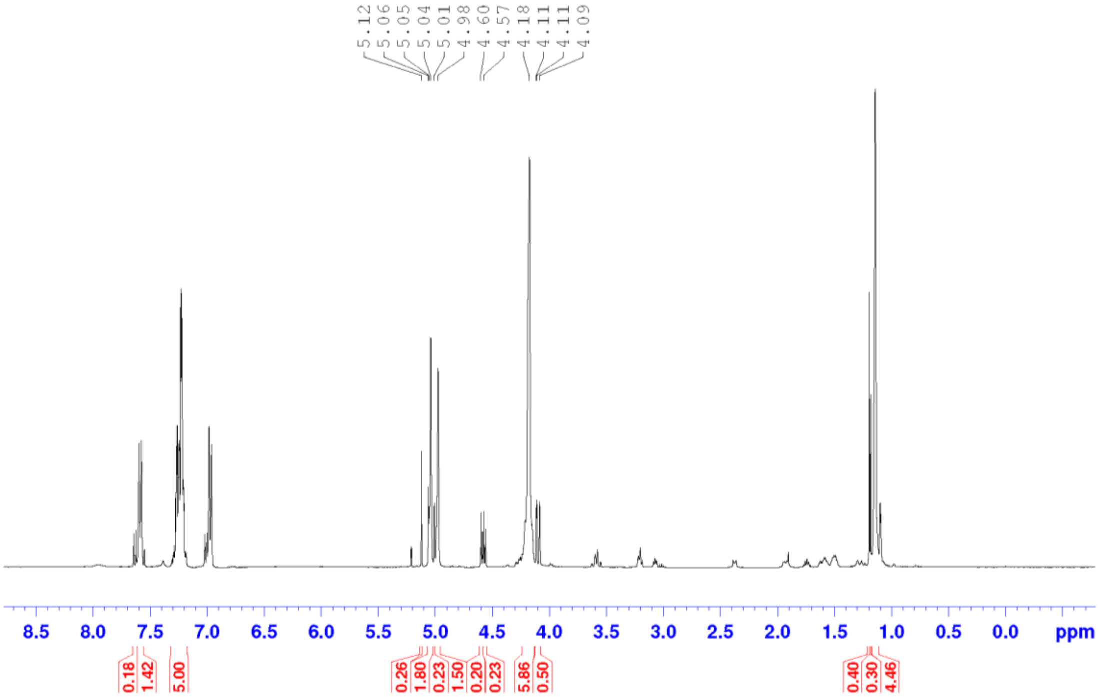

Figure S15a. The ^{1}H NMR spectrum (CD_2Cl_2) of the polymerization of 50% ArI-TMC and 50% Bn-TMC at 60 minutes of reaction time.

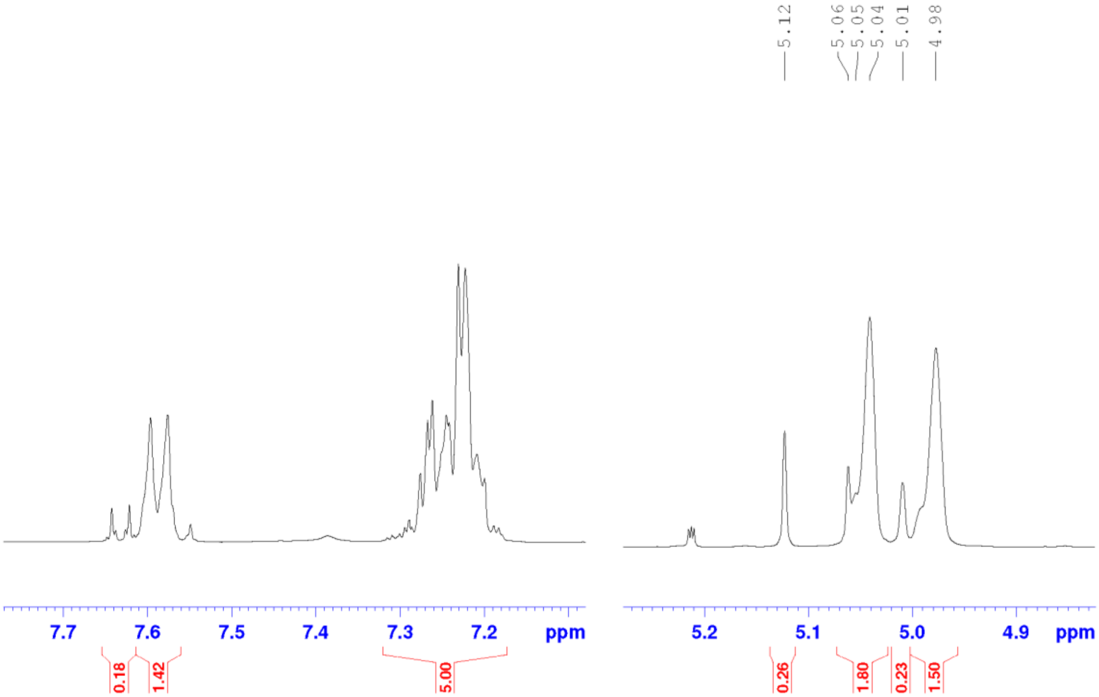

Figure S15b. Expansions of the ^{1}H NMR spectrum (CD_2Cl_2) at 60 minutes of reaction time. The percent conversion of ArI-TMC was 89% and the percent conversion of Bn-TMC was 87%.

III. Triphenylphosphine oxidation

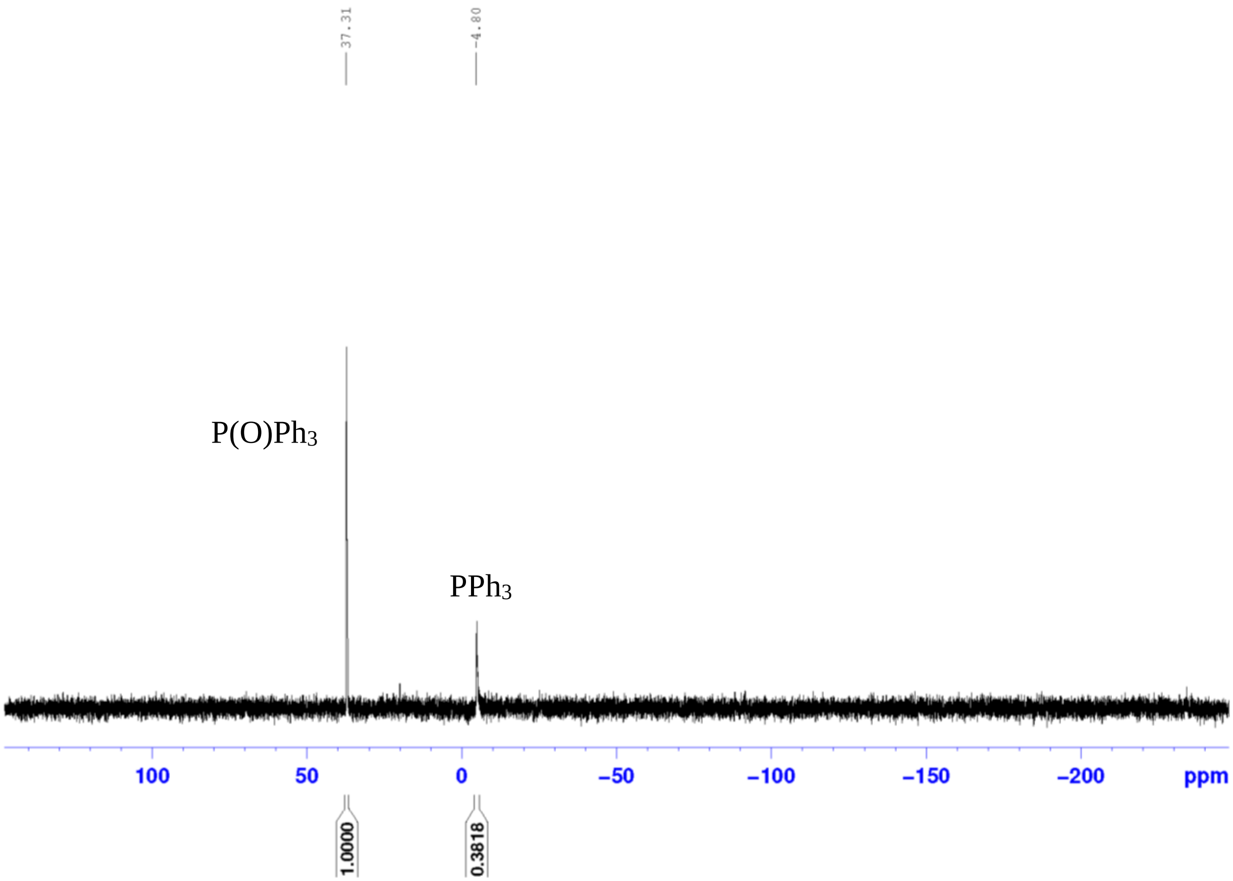

Figure S16. The ^{31}P NMR spectrum ($CDCl_3$) of the oxidation of triphenylphosphine to triphenylphosphine oxide using the iodosyl copolymer. The two species were verified by known standards of triphenylphosphine and triphenylphosphine oxide.

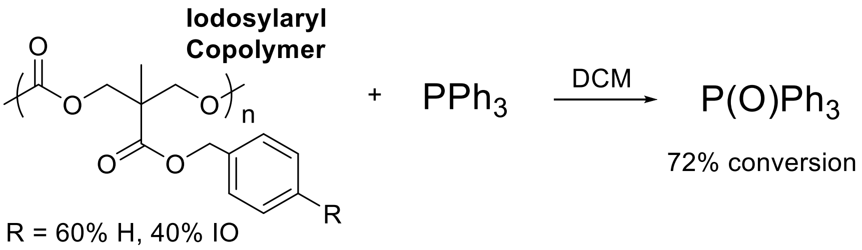

Supplement: Supplementary file 1 [file polymers-15-02052-s001.zip › polymers-2313142-SI.pdf]
